# Supplementary material for: When population growth intensifies intergroup competition, female colobus monkeys free-ride less
Source: Sci Rep. 2024 Jun 21;14:14363. doi: 10.1038/s41598-024-64188-0 (PMC11192885; doi:10.1038/s41598-024-64188-0)
Supplement: Supplementary file 1 — Supplementary Information 1. [file 41598_2024_64188_MOESM1_ESM.docx]

**When population growth intensifies intergroup competition, female colobus monkeys free-ride less**

T. Jean M. Arseneau-Robar*^1^, Julie A. Teichroeb^1^, Andrew J. J. Macintosh^2^, Tania L. Saj^3^, Emily Glotfelty^4^, Sara G. Lucci^4^, Pascale Sicotte^5^ and Eva C. Wikberg^4^

^1^ Department of Anthropology, University of Toronto Scarborough, Toronto, Canada

^2^ Wildlife Research Center, Kyoto University, Kyoto, Japan

^3^ Department of Anthropology, University of Calgary, Calgary, Canada

^4^ Department of Anthropology, University of Texas San Antonio, San Antonio, USA

^5^ Department of Biology, Concordia University, Montreal, QC, Canada

* Corresponding authors: T. Jean M. Arseneau-Robar; Eva C. Wikberg

Email: [arseneau.jean.m@gmail.com](mailto:arseneau.jean.m@gmail.com); [eva.wikberg@utsa.edu](mailto:eva.wikberg@utsa.edu)

**Supplementary Methods**

***Estimating food availability for each group over time***

We created an aggregated food-availability index (FAI) that approximated the availability of monopolizable foods in the home range of each group, during each study period. To estimate the annual home range for each study group each study period, we used the location of the centre-of-mass of the group every 30 minutes. We used the ‘adehabitatHR’ package [1] to estimate the utilization distribution using the kernel density estimate approach [2], setting the smoothing parameter to the reference bandwidth. Home range boundaries were derived from the 95% contour of the utilization distribution. We used the observed diet of each study group [3,4, Wikberg unpublished data], during each study period, to create a list of 28 ‘important food-species’ that comprised at least 5% of the annual diet of at least one group. We mapped the location of each individual tree, having a diameter at breast-height (DBH) of at least 40 cm, of these important food-species across the study area in 2000-2001 [3]. We used this detailed tree map to determine the total DBH for the important food-species within each annual home range. We used DBH because this metric correlates well with canopy size and overall tree productivity [5]. During our monthly phenology sampling, the abundance of flowerbuds and flowers, unripe and ripe fruits, unripe and ripe seedpods, and young and mature leaves were estimated for three to seven (mean = 5) representative trees of these important food species [6]. Observers gave each plant part a score from 0 to 4 (0 = none, 1 = 1% to 25%, 2 = 26% to 50%, 3 = 51% to 75%, 4 = 76% to 100% canopy cover) to index the canopy cover of that plant part; the maximum total score within each category (e.g., flowers, fruits, seeds, leaves) was 4 [3]. We calculated the average monthly phenology score for each plant part, of each species, and multiplied these average scores by the total DBH of that species in each home range, to get an index of food availability (FAI) for each plant part of each species, for each group [7,8]. We then created an aggregated FAI by summing the FAIs of flowerbuds, flowers, unripe fruits, ripe fruits, unripe seedpods, ripe seedpods and young leaves for all important food-species. Because these high-quality foods are also those that tended to have more patchy distributions, they are also expected to be those that would incite intergroup contest competition.


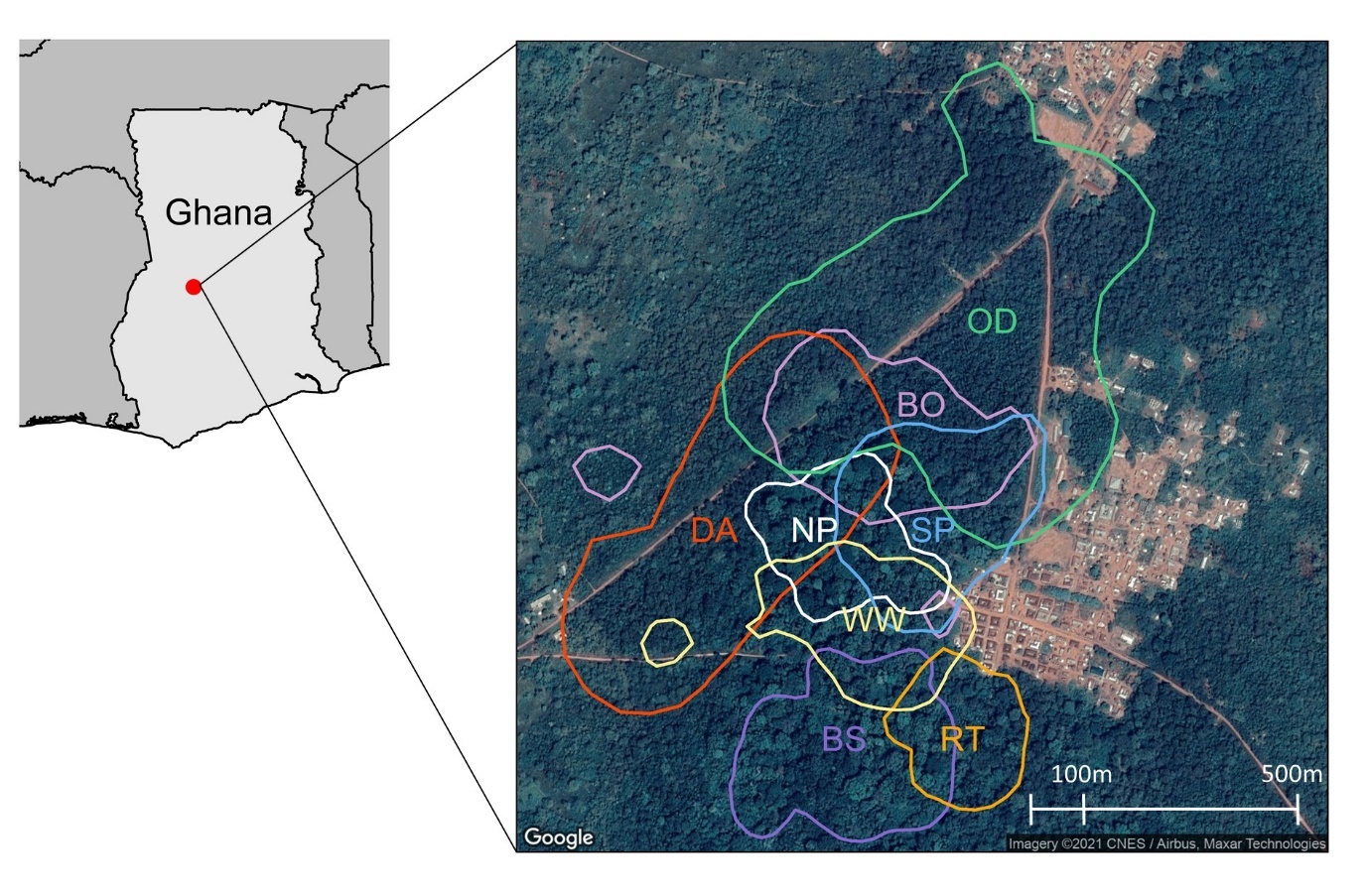


**ESM Figure 1.** Home ranges of the eight study groups of ursine colobus monkeys at Boabeng-Fiema Monkey Sanctuary in Ghana, during the 2008-2009 study period. Map created by the authors using R [9].


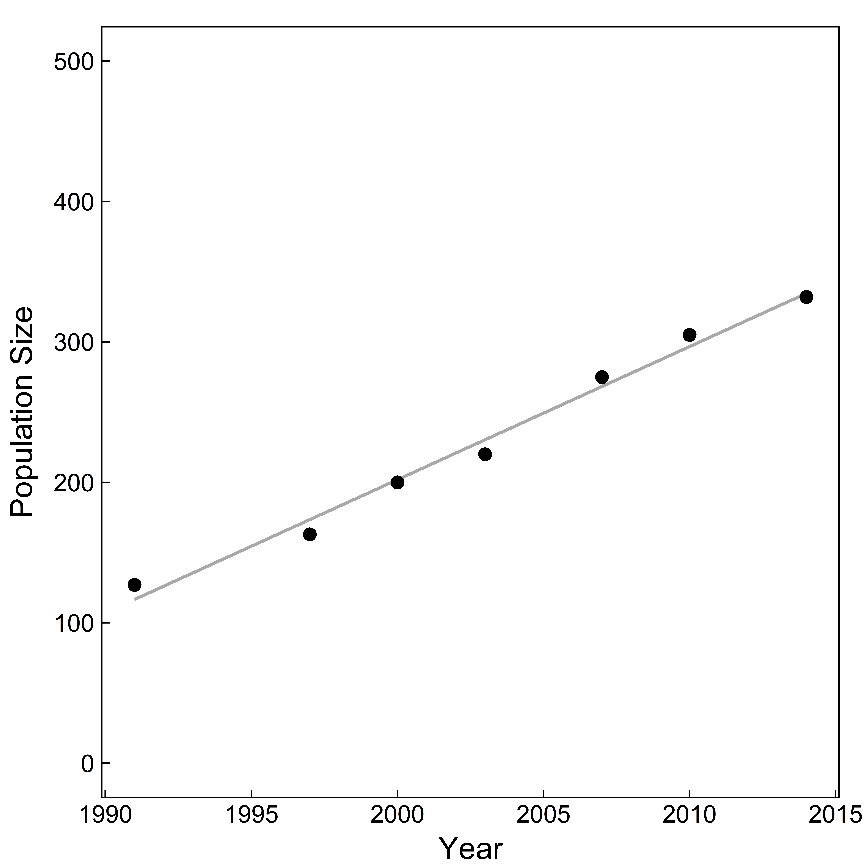


**ESM Figure 2.** Size of the population of ursine colobus monkeys living at the Boabeng-Fiema Monkey Sanctuary in Ghana between 1991 and 2014.


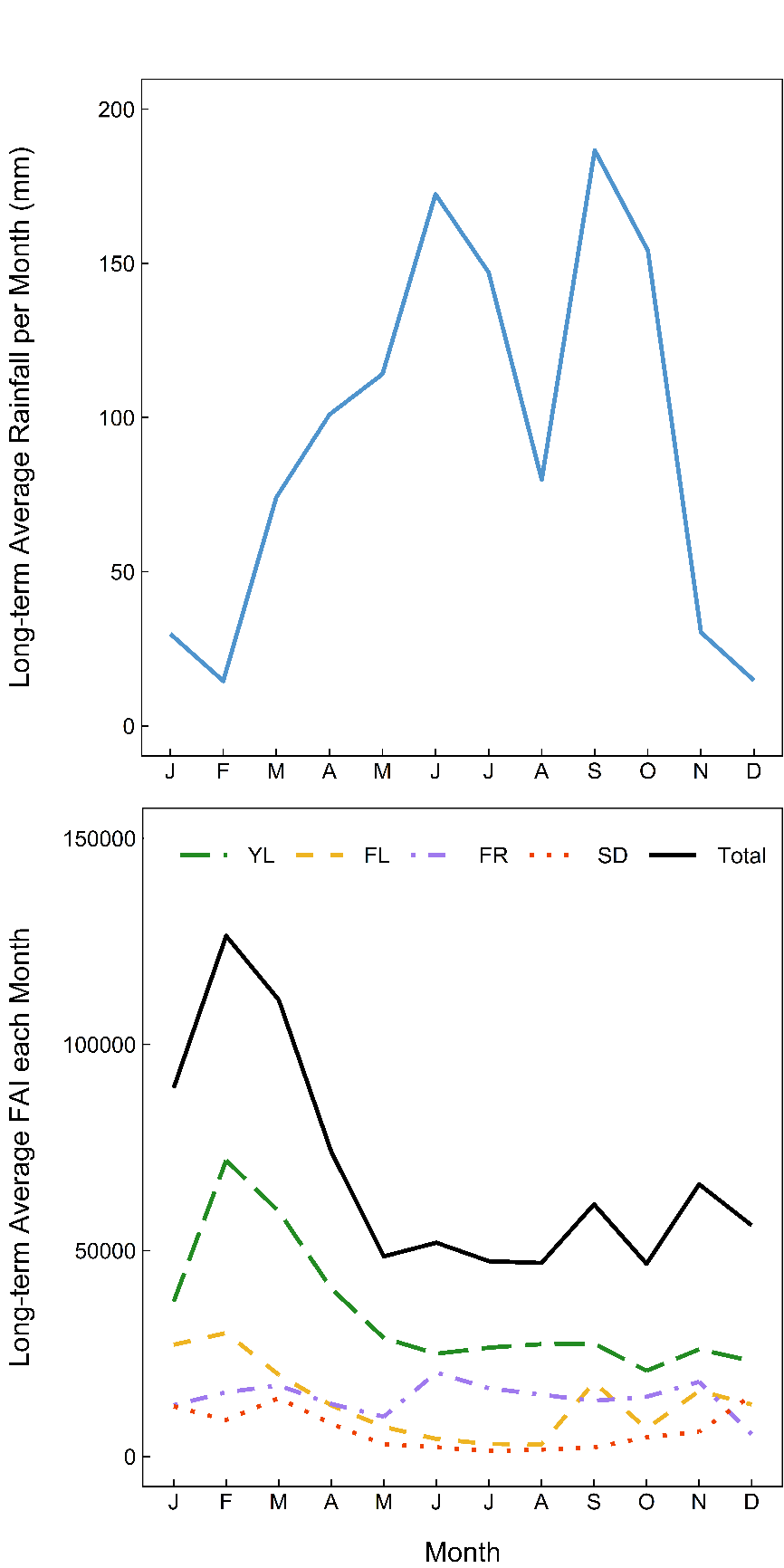


**ESM Figure 3.** Seasonal variation in rainfall and the availability of young leaves, flowers, fruits and seeds in the study area.

**References**

1. Calenge, C. The package ‘adehabitat’ for the R software: a tool for the analysis of space and habitat use by animals. *Ecol. Mod.* **197**, 516-519. <https://doi.org/10.1016/j.ecolmodel.2006.03.017> (2006).

2. Worton, B. J. Using Monte Carlo simulation to evaluate kernel-based home range estimators. *J. Wildl. Manag*. **59**, 794-800. <https://doi.org/10.2307/3801959> (1995).

3. Saj, T. L., & Sicotte, P. Predicting the competitive regime of female *Colobus vellerosus* from the distribution of food resources. *Int. J. Primatol.* **28**, 315–336. https://doi.org/10.1007/s10764-007-9124-x (2007).

4. Teichroeb, J. A., & Sicotte, P. Test of the ecological-constraints model on ursine colobus monkeys (*Colobus vellerosus*) in Ghana. *Am. J. Primatol*. **71**, 49-59. https://doi.org/10.1002/ajp.20617

5. Chapman, C. A., Chapman, L. J., Wrangham, R., Hunt, K., Gebo, D., & Gardner, L. Estimators of fruit abundance of tropical trees. *Biotropica* **24**, 527-531. <https://doi.org/10.2307/2389015> (1992).

6. Saj, T.L., Sicotte, P. Scramble Competition Among *Colobus vellerosus* at Boabeng-Fiema, Ghana. *Int. J. Primatol.* **28**, 337–355. <https://doi.org/10.1007/s10764-007-9125-9> (2007).

7. Dasilva, G. L. Diet of *Colobus polykomos* on Tiwai Island: Selection of food in relation to its seasonal abundance and nutritional quality. *Int. J. Primatol.* **15**, 655–680. https://doi.org/10.1007/BF02737426 (1994).

8. Fashing, P. J. The behavioural ecology of an African colobine monkey: diet, range use, and patterns of intergroup aggression in eastern black and white colobus monkeys (*Colobus guereza*). PhD Dissertation, Columbia University.

9. R Core Team. R: a language and environment for statistical computing. R Foundation for Statistical Computing, Vienna, Austria. See <https://www.r-project.org/>. (2017).
