# Supplementary material for: When population growth intensifies intergroup competition, female colobus monkeys free-ride less
Source: Sci Rep. 2024 Jun 21;14:14363. doi: 10.1038/s41598-024-64188-0 (PMC11192885; doi:10.1038/s41598-024-64188-0)
Supplement: Supplementary file 3 — Supplementary Information 3. [file 41598_2024_64188_MOESM3_ESM.pdf]

R Code for:

When population growth intensifies intergroup competition, female colobus monkeys free-ride less

T. Jean Arseneau-Robar*[1], Julie A. Teichroeb[1], Andrew J. J. Macintosh[2], Tania L. Saj[3], Emily Glotfelty[4], Sara Lucci[4], Pascale Sicotte[5] and Eva C. Wikberg*[4]

[1] Department of Anthropology, University of Toronto Scarborough, Toronto, Canada

[2] Wildlife Research Center, Kyoto University, Kyoto, Japan

[3] Department of Anthropology, University of Calgary, Calgary, Canada

[4] Department of Anthropology, University of Texas San Antonio, San Antonio, USA

[5] Department of Biology, Concordia University, Montreal, QC, Canada

```
setwd("")  # set the working directory


library(ggplot2)     # for making basic graphs
library(gridExtra)  # making multi-plot graphs (Fig 1A and 1B in one plot)
library(lme4)      # binomial and poisson GLMMs, bootstrapped CIs
library(MuMIn)     # R2 value for GLMMs
library(DHARMa)    # assessing model performance
library(car)       # correlations and VIF

##########################################################################
########################################
##### OPEN DATA FILE
##########################################################################
##################
BGE_final <- read.csv("IGE_final_dataset.csv")


# subset out NAs and make datasets for models
BGE_clean_2 <- subset(BGE_final, BGE_final$CurrFAI_scaled != "NA")
BGE_clean_3 <- subset(BGE_clean_2, BGE_clean_2$NumAF_scaled != "NA")
BGE_clean_4 <- subset(BGE_clean_3, BGE_clean_3$NoBGE_F_Part != "NA")
BGE_clean_5 <- subset(BGE_clean_4, BGE_clean_4$NoBGE_M_Part != "NA")
MI_M_data <- subset(BGE_clean_3, BGE_clean_3$NoMI_No_M_Part >=0)
```

```
MI_F_data <- subset(BGE_clean_3, BGE_clean_3$NoMI_No_F_Part >=0)

# scale any variables not already scaled
BGE_clean_5$BGE_rate_scaled <- scale (BGE_clean_5$BGE_rate)


###########################################################################
################################################
##### RATES OF WHOLE-GROUP ENCOUNTERS
###########################################################################
#########
BGE_rate_model <- glmer(NoBGE ~
                  Rainfall_3_scaled +
                  PopDensity_scaled +
                  CurrFAI_scaled +
                  NumAF_scaled +
                  NumAM_scaled +
                  offset(log(ContactHrs)) +
                  (1|Group),
                 family= poisson,  data= BGE_clean_3)
summary(BGE_rate_model)

#### LRT instead of Wald p-values
drop1(BGE_rate_model, test="Chisq")

NULLmodel <-  glmer (NoBGE ~  offset(log(ContactHrs)) + (1|Group),
              family= poisson, data= BGE_clean_3,
              (control=glmerControl(optimizer="bobyqa", optCtrl=list(maxfun=100000))))
anova(BGE_rate_model, NULLmodel )

#### check for overdispersion
simulationOutput <- simulateResiduals(fittedModel   = BGE_rate_model)
plot(simulationOutput)


###########################################################################
################################################
#### RATES OF MI
###########################################################################
###############################
MI_rate_model <- glmer(TotalNoMI ~
                 Rainfall_3_scaled +
                 PopDensity_scaled +
                 CurrFAI_scaled +
```

```
                  NumAF_scaled +
                  SM_YN +
                  PopDensity_scaled*SM_YN +
                    offset(log(ContactHrs)) +
                    (1|Group) ,
                  family= poisson,  data= BGE_clean_3,
                  (control=glmerControl(optimizer="bobyqa", optCtrl=list(maxfun=100000))))
summary(MI_rate_model)

#### LRT instead of Wald p-values
drop1(MI_rate_model, test="Chisq")

MIrate_NULLmodel <-  glmer (TotalNoMI ~  offset(log(ContactHrs)) + (1|Group) ,
                    family= poisson, data= BGE_clean_3,
                    (control=glmerControl(optimizer="bobyqa", optCtrl=list(maxfun=100000))))
anova(MIrate_NULLmodel, MI_rate_model)


#### model diagnostics
simulationOutput <- simulateResiduals(fittedModel   = MI_rate_model)
plot(simulationOutput)


testDispersion(simulationOutput) #option 1 - no sig dispersion
simulationOutput2 <- simulateResiduals(fittedModel = MI_rate_model, refit = T, n = 20)
testDispersion(simulationOutput2) # option 2 - no sig dispersion
# although sig, the dispersion parameter is not actually very high
# A word of warning that applies also to all other tests that follow: significance in
hypothesis tests
# depends on at least 2 ingredients: strenght of the signal, and number of data points.
Hence, the p-value
# alone is not a good indicator of the extent to which your residuals deviate from
assumptions. Specifically,
# if you have a lot of data points, residual diagnostics will nearly inevitably become
significant, because
# having a perfectly fitting model is very unlikely. That, however, doesn't neccessarily mean
that you need
# to change your model. The p-values confirm that there is a deviation from your null
hypothesis. It is,
# however, in your discretion to decide whether this deviation is worth worrying about. If
you see a
# dispersion parameter of 1.01, I would not worry, even if the test is significant. A significant
value of 5,
# however, is clearly a reason to move to a model that accounts for overdispersion.
```

```
#######################################################################
##########################################
#### MALE PARTICIPATION IN WHOLE-GROUP ENCOUNTERS
################################################################
male_part_model <- glmer(cbind(NoBGE_M_Part, NoBGE_No_M_Part) ~
                  Rainfall_scaled +
                  PopDensity_scaled +
                  CurrFAI_scaled+
                  NumAF_scaled +
                  SM_YN +
                  CurrFAI_scaled * NumAF_scaled +
                  (1|Group) ,
                 family= binomial,  data= BGE_clean_5,
                 (control=glmerControl(optimizer="bobyqa", optCtrl=list(maxfun=100000))))
summary(male_part_model)

#### LRT instead of Wald p-values
drop1(male_part_model, test="Chisq")

NULLmodel_malepart <-  glmer (cbind(NoBGE_M_Part, NoBGE_No_M_Part) ~ (1|Group) ,
                                          family= binomial,  data= BGE_clean_5,
                                          (control=glmerControl(optimizer="bobyqa",
optCtrl=list(maxfun=100000))))
anova(NULLmodel_malepart, male_part_model)

### check for overdispersion
mAGG_simulationOutput <- simulateResiduals(fittedModel   = male_part_model)
plot(mAGG_simulationOutput)




#######################################################################
#########################################
#### FEMALE PARTICIPATION IN WHOLE-GROUP ENCOUNTERS
#############################################################
female_part_model <- glmer(cbind(NoBGE_F_Part, NoBGE_No_F_Part) ~
                   Rainfall_scaled +
```

```
                    PopDensity_scaled +
                    CurrFAI_scaled +
                    NumAF_scaled +
                    SM_YN +
                    CurrFAI_scaled * NumAF_scaled +
                     (1|Group),
                    family= binomial,  data= BGE_clean_5,
                    (control=glmerControl(optimizer="bobyqa", optCtrl=list(maxfun=100000))))
summary(female_part_model)

#### LRT instead of Wald p-values
drop1(female_part_model, test="Chisq")

NULLmodel_femalepart <-  glmer (cbind(NoBGE_F_Part, NoBGE_No_F_Part) ~ (1|Group),
                      family= binomial,  data= BGE_clean_5,
                      (control=glmerControl(optimizer="bobyqa", optCtrl=list(maxfun=100000))))
anova(NULLmodel_femalepart, female_part_model)

##### Check model diagnostics
fAGG_simulationOutput <- simulateResiduals(fittedModel   = female_part_model)
plot(fAGG_simulationOutput) # plot 1 looks good as resid follow line pretty well (no S
shape) and are not




########################################################################
##############################################
##### MALE PARTICIPATION IN MALE INCURSIONS
########################################################################
###
male_partMI_model <- glmer(cbind(NoMI_M_Part, NoMI_No_M_Part) ~
                    Rainfall_scaled +
                    PopDensity_scaled +
                    CurrFAI_scaled +
                    NumAF_scaled +
                    SM_YN +
                   (1|Group) ,
                 family= binomial,  data= BGE_clean_3,
                 (control=glmerControl(optimizer="bobyqa", optCtrl=list(maxfun=100000))))
summary(male_partMI_model)
```

```
#### LRT instead of Wald p-values
drop1(male_partMI_model, test="Chisq")

NULLmodel_maleMIpart <-  glmer (cbind(NoMI_M_Part, NoMI_No_M_Part) ~ (1|Group),
                    family= binomial,  data= BGE_clean_3,
                    (control=glmerControl(optimizer="bobyqa", optCtrl=list(maxfun=100000))))
anova(NULLmodel_maleMIpart, male_partMI_model)


##### Check model diagnostics
mAGG_MI_simulationOutput <- simulateResiduals(fittedModel   = male_partMI_model)
plot(mAGG_MI_simulationOutput) # plot 1 looks good as resid follow line pretty well (no S shape) and are not




##########################################################################
######################################################
##### FEMALE PARTICIPATION IN MALE INCURSIONS
##########################################################################
#######
female_partMI_model <- glmer(cbind(NoMI_F_Part, NoMI_No_F_Part) ~
                    Rainfall_scaled +
                    PopDensity_scaled +
                    CurrFAI_scaled +
                    NumAF_scaled +
                    SM_YN +
                    (1|Group),
                    family= binomial,  data= BGE_clean_3,
                  (control=glmerControl(optimizer="bobyqa", optCtrl=list(maxfun=100000))))
summary(female_partMI_model)

#### LRT instead of Wald p-values
drop1(female_partMI_model, test="Chisq")

##### Compare to the null model with random effects only to assess if the model is actually good (explains significant amount of variability in the data)
NULLmodel_femaleMIpart <-  glmer (cbind(NoMI_F_Part, NoMI_No_F_Part) ~ (1|Group),
                     family= binomial,  data= BGE_clean_3,
                     (control=glmerControl(optimizer="bobyqa", optCtrl=list(maxfun=100000))))
anova(NULLmodel_femaleMIpart, female_partMI_model)
```

```
##### Check model diagnostics
fAGG_MI_simulationOutput <- simulateResiduals(fittedModel   = female_partMI_model)
plot(fAGG_MI_simulationOutput)


########################################################################
########################################
##### CONFIDENCE INTERVALS
########################################################################
#############
confint.merMod(BGE_rate_model, method="profile")
confint.merMod(MI_rate_model, method="profile")
confint.merMod(male_part_model, method="profile")
confint.merMod(female_part_model, method="profile")
confint.merMod(male_partMI_model, method="profile")
confint.merMod(female_partMI_model, method="profile")


########################################################################
########################################
##### testing VIF
########################################################################
######################
vif(BGE_rate_model)
vif(MI_rate_model)
vif(male_part_model)
vif(female_part_model)
vif(male_partMI_model)
vif(female_partMI_model)


########################################################################
########################################
##### R2 value for GLMMs from Nakagawa et al. 2017  --- use delta
##############################################
r.squaredGLMM(BGE_rate_model)
r.squaredGLMM(MI_rate_model)
r.squaredGLMM(male_part_model)
r.squaredGLMM(female_part_model)
r.squaredGLMM(male_partMI_model)
r.squaredGLMM(female_partMI_model)
```
